# Editor's Letter-Box

**Published:** 1893-07-29

**Authors:** J. Brendon Curgenven

**Affiliations:** Teddington Hall


### EDITOR'S LETTER-BOX.

[Our correspondents are reminded that prolixity is a great bar to publication, and that brevity of style and conciseness of statement greatly facilitate early insertion.]

Sir,—In my letter I said "Sanitus," not "Sanitas," oil, for I presume it was the "Sanitus" fluid which Dr. Rogers used. I have tested the properties of "Sanitas" oil, and have found it totally unfit for inunction on account of the amount of resin in it, and I enclose paper on which I tested it in 1891 for your inspection; I enclose also paper on which I have to-day dropped the "Oleusaban" eucalyptus, and you will see that it has evaporated without leaving the slightest stain. My evidence and that of others on the use of the "Oleusaban" is to be found in my papers and letters in the medical journals. Let Mr. Kingzett support his assertions with an equal array of facts.—Yours truly,

J. Brendon Curgenven.

Teddington Hall, July 17th, 1893.

